# Comparative transcriptomics approach in elucidation of carotenoid biosynthesis regulation in grains of rice (*Oryza sativa* L.)

**DOI:** 10.1038/s41598-018-38233-8

**Published:** 2019-02-07

**Authors:** Upasna Chettry, Nikhil K. Chrungoo, Kirti Kulkarni

**Affiliations:** 1 North Eastern Hill University, Department of Botany, Shillong, 793022 India; 2 Bionivid Technologies, Bangalore, India

## Abstract

Estimation of phytoene, lycopene, β-carotene, lutein, and zeaxanthin in grains of white, brown and purple cultivars of rice revealed marked differences in the levels of these carotenoid intermediates amongst the cultivars. Grains of white rice did not show any significant accumulation of carotenoid intermediates at any stage of development. On the other hand, grains of the purple cultivar accumulated 49.16 ± 5 µg of β-carotene, 28.89 ± 3.2 µg of lutein and 34.65 ± 4.6 µg of zeaxanthin per gm of grain fresh weight. In addition to PSY1, higher expression of βLCY than εLCY appears to be an important factor in determining the flux of pathway towards synthesis of β-β branch carotenoids in purple rice. This cultivar showed a higher fold change in carotenoid precursors during transition from milky to doughing stages and an enhanced flux of lycopene towards β-carotene during grain maturation. Our results indicate that higher level of carotenoids in purple rice is a consequence of higher expression of genes involved in pyruvate metabolism as well as those involved in carotenoid biosynthesis such as PSY1, PDS and β-LCY. Co-expression networking revealed a strong positive relationship between the expression profiles of genes involved in carotenoid biosynthesis and genes coding for geranylgeranyl transferase type II, glutathione S-transferase, DnaJ and SET domain containing proteins as well as MADS26 and R2R3MYB family of transcription factors.

## Introduction

As a staple food of bulk of the population of Southeast Asia, rice grains contribute significantly towards meeting nutritional requirements of the people of this region. However, low levels of calcium, iron, thiamine and riboflavin and near absence of β-carotene makes rice as one of the low nutrient staple foods. Thus, exclusive reliance on rice as a primary food leads to vitamin A deficiency, a serious public health problem. Strategies to boost the content of β-carotene, the precursor for vitamin A, in endosperm of rice grains have focused around expression of heterologous PSY and the bacterial CRTI, which replaced several consecutive reactions that are required for conversion of phytoene to lycopene^1^. Bai *et al*.^2^ and Gayen *et al*.^3^ have, however, identified pyruvate flux, isoprenoid precursor pool and metabolic sink as additional bottlenecks in carotenoid biosynthesis and accumulation in rice. With a limited knowledge of the regulatory controls involved in carotenoid biosynthesis in rice, availability of mutants with altered carotenoid biosynthetic pathway would have been an important milestone in understanding carotenoid biosynthesis in this crop. While such a collection of mapped and biochemically characterized mutants is available in other grain crops such as maize^4,5^, not many mutants of this type have been identified in rice. Wurtzel *et al*.^6^ presented the first evidence of a vivipary mutation 84NMEMdr2 and 90KHEMdr1in rice which also caused albino phenotype. Besides vivipary, the mutation also conferred a block in the conversion of phytoene to lycopene, thereby hindering biosynthesis of downstream intermediates of the carotenoid pathway in grains. Low expression of phytoene desaturase or impaired biosynthesis of plastoquinones was suggested as the be main reason for block in the conversion of phytoene to lycopene. Fang *et al*.^7^ have identified 4 *phs* mutants in rice which had mutations in PDS, ZDS CRITSO and βLCY genes. The mutants showed viviparous phenotype with altered carotenoid composition as well as impaired plastid assembly. While the mutations confirmed the role of PDS, ZDS CRITSO and βLCY in carotenoid biosynthesis, they did not provide any information on the regulatory controls involved in carotenoid biosynthesis in rice. Pigmented cultivars, which are rich in bioactive compounds including anthocyanins and carotenoids^8–14^, provide an alternative for analyzing the regulatory factors involved in carotenoid biosynthesis in rice. To address the challenge of understanding the regulators of carotenoid biosynthesis in rice, we used rice grains from Northeast India varying in the pericarp color (white, brown and purple) as contrasting phenotypes for deep transcriptome sequencing at different developmental stages.

## Material and Methods

### Plant Material

**Figure 1:**
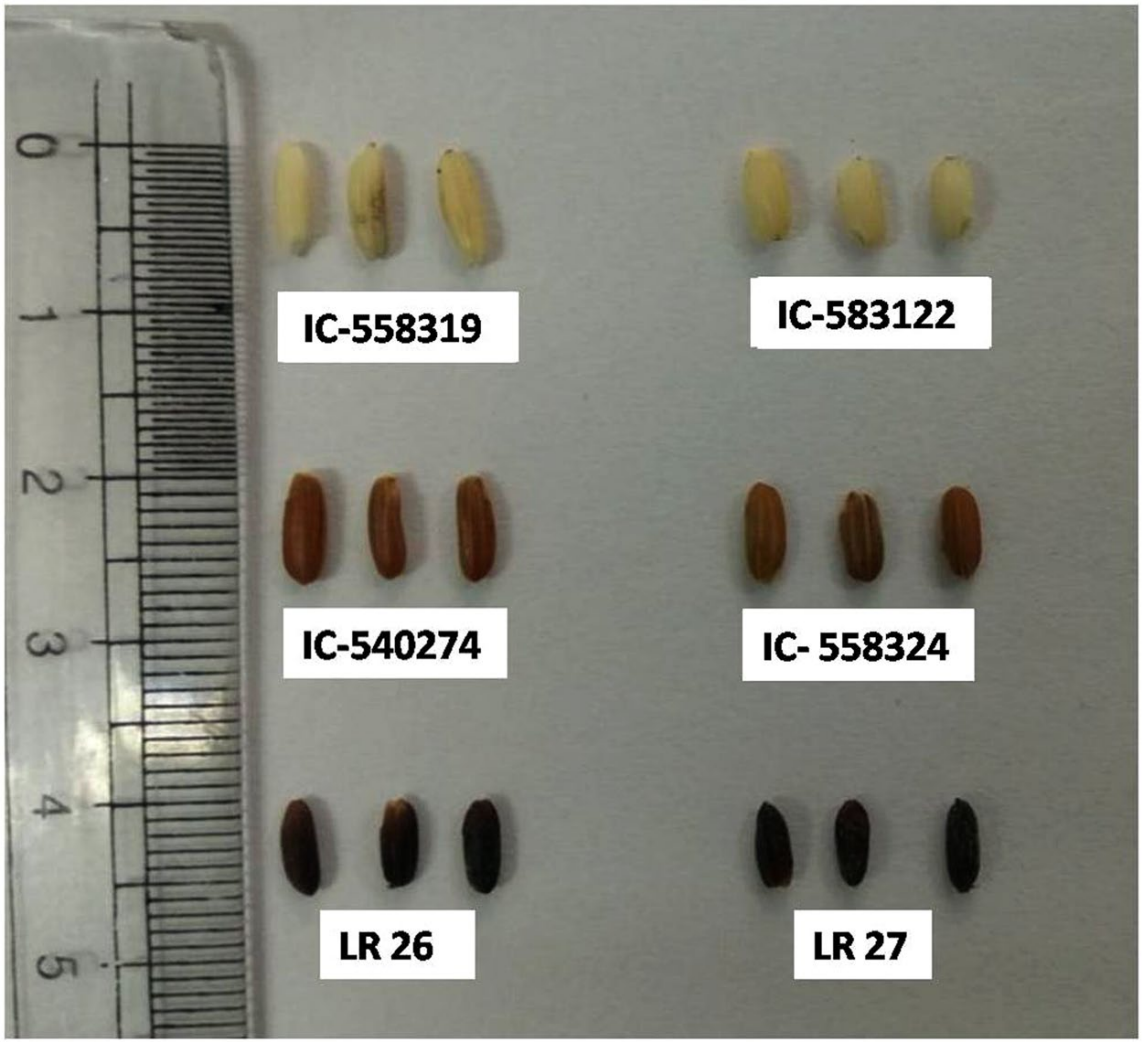
Grains of different accessions of rice from North East India studied in the present investigation showing variations in the colour of their pericarp. White(IC-558319, IC-583122), Brown(IC-540274, IC-558324), Purple(LR 26, LR 27).

Grains of white (IC-583122, IC-558319), brown (IC-540274, IC-558324) and purple (LR 26, LR 27) cultivars of rice (*Oryza sativa* L.) were procured from National Bureau of Plant Genetic Resources (NBPGR) and Central Agricultural University, Barapani, Shillong (Fig. 1). Seedlings of each accession were raised to maturity in an environment controlled green house under a 12 hour photoperiod and temperature of 26 ± 2 °C during day and 22 ± 2 °C during night. The grains were harvested at milky (15 DAP), doughing (25 DAP) and mature (40 DAP) stage of development and dehulled. The dehulled grains were cleaned and stored at −80 °C.

### Targeted estimation of carotenoid intermediates

Estimation of phytoene, lycopene, β-carotene, lutein, and zeaxanthin was carried out in whole grains of the six cultivars of rice harvested at milky (15DAP), doughing (25DAP) and mature (40DAP) stages of grain development according to Panfili *et al*.^15^ with certain modifications. A suitable mass of the harvested tissue from each cultivar was crushed to fine powder under liquid nitrogen in a pre-chilled pestle and mortar. A defined mass of the powdered tissue was suspended in three volumes of 95% ethanol containing 0.1% ascorbic acid. 1/3^rd^ volume of cold 1% sodium chloride was added to the mixture and the entire solution was vortexed and incubated in a water bath at 85 °C for 10 minutes. Saponification was carried out by adding 1/3 volume of 80% potassium hydroxide and heating in a water bath at 85 °C for 20 minutes. The suspension was mixed with equal volume of n-hexane-ethyl acetate (9:1) and the organic phase was transferred to a fresh tube. The aqueous phase was mixed again with equal volume of n-hexane-ethyl acetate (9:1) and the organic phase collected. The organic phases were pooled, dried under nitrogen gas at 40 °C and re-dissolved in 500 μl of methanol:acetonitrile (40:60) for chromatography on Spherisorb ODS2 C18 reverse phase column (25 × 3.2 mm) at a flow rate of 0.45 ml min^−1^ using methanol:acetonitrile:2-propanol (40:58:2) as the eluent. Each sample was filtered through a 0.4 µm nylon filter (SFNY25X) (Axiva) prior to injection into the column. The extinction values for each peak were monitored at 450 nm with phytoene, lycopene, β-carotene, lutein, and zeaxanthin as the reference standards. The concentration of a carotenoid in a fraction was calculated from the peak areas with reference to the peak area determined for the eluted reference standard. All analyses were carried out with eight biological and three technical replicates. The data was statistically tested for significance of variations by analysis of variance (ANOVA) at P 0.05.

### Transcriptome library preparation and sequencing

Total RNA was extracted from grains of white (IC-583122), brown (IC-540274) and purple (LR 26) cultivars of rice harvested at milky (15 DAP), doughing (25 DAP) and mature (40 DAP) stages of grain development, using Trizol reagent (Invitrogen) with certain modification. The quality of RNA was assessed with Agilent 2100 Bioanalyzer. RNA samples with RIN value ≥7.0 were subjected to deep transcriptome sequencing using Illumina HiSeq2000 sequencing platform as per manufacturer’s protocol.

### *In-silico* analysis of transcriptome data

#### Quality Control Analysis and Alignment

The raw reads were subjected to quality check using “NGS-QC tool kit v2.3”^16^. Reads having 70% of the bases with a “Phred” score of >20 were considered as high quality and used for further analysis. Sample-wise high-quality reads were aligned to the rice reference genome (http://rice.plantbiology.msu.edu/) using “TopHat v2.0.3”. The aligned reads were subsequently used to create a reference annotation based transcript (RABT) assembly to identify uniquely aligned reads in each sample.

#### Quantification and differential expression of transcripts

We used “Cufflinks v2.2.0” to identify the differentially expressed transcripts across the cultivars at each stage of grain development and within cultivars for different stages of grain development. The assembled samples were combined using “Cuffmerge v2.2.0” and subjected to “Cuffcompare v2.2.0” for comparison with transcripts available in msu 7 database. The results obtained after data analysis by Cuffcompare were subjected to “featureCounts v1.5.0” to obtain the read count of all samples. Differences in gene expression across different stages of grain development within cultivars and at each stage of grain development across the cultivars were identified using “DESEq2 1.2.5R”. The thresholds for P-value and FDR (adjusted P-value or Q-value) were set to 0.001 for identifying significant differences in expression. Gene Ontology (GO) was derived from the msu 7 rice genome database and the pathways were identified using Kyoto Encyclopedia of Genes and Genomes (KEGG). The GO analysis of novel transcripts and highly expressed genes was plotted using the WEGO (Web Gene Ontology Annotation Plot) program (http://wego.genomics.org.cn/cgi-bin/wego/index.pl)^17^. Transcription factors were identified from the data sets using the annotation from Database of Rice Transcription Factors and Plant Transcription Factor Database.

#### Pathway analysis and BAN (Biological Analysis Network) modelling

The data obtained on Gene Ontologies, Pathways and DEGs was used as input for pathway analysis and biological analysis network modeling using “BridgeIsland Software” (Bionivid Technology, India). The output file from Bridge Island was fed to “CytoScape V 2.8” to derive molecular interaction networks.

#### Co-expression networking

For a comparative analysis of transcription factors co-expressing with the carotenoid biosynthesis pathway across the white, brown and purple cultivars of rice, a correlation analysis of genes involved in carotenoid biosynthesis and transcription factors was carried out using “CORREL” function in “EXCEL2013”following Zhang *et al*.^18^ and Ye *et al*.^19^. In order to exclude false positives, structural genes and transcription factors with FPKM value of ≥5.0 in at least one of the three stages of grain filling were selected. Transcription factors with correlation coefficient values of ≥0.8 were considered to have an expression that was significantly correlated with the expression of carotenoid biosynthetic pathway. All data has been expressed as mean of independent replicates ±SD at P0.05. The statistical significance of variations has been calculated using student’s *t*-test.

### Validation of gene expression

Validation of the expression profiles of PSY1, PDS, β-LCY, ε-LCY, BCH, hexokinase, pyruvate kinase, fructose-1,6bisphosphatase, geranylgeranyl transferase, 9- cis-epoxycarotenoid dioxygenase, MADS26 and MYB genes was carried out by real-time PCR using gene-specific primers listed in Table s1. Each reaction was carried out with three independent biological replicates and three technical replicates. The relative expression levels for each gene were normalized to the expression level of actin (internal reference gene), which was calculated from cycle threshold values using the 2^− Ct^ method according to Livak and Schmittgen^20^.

## Results

### Targeted estimation of carotenoid intermediates

Estimation of phytoene, lycopene, β-carotene, lutein and zeaxanthin in whole grains of white (IC-583122, IC-558319), brown (IC-540274, IC-558324) and purple (LR 26, LR 27) cultivars of rice harvested at milky (15DAP), doughing (25DAP) and mature (40DAP) stages of grain development revealed significant differences in the levels of these carotenoid intermediates amongst the cultivars. Except for marginal levels of lutein in mature grains of IC-583122, no other carotenoid intermediate was detected in grains of white cultivars at any stage of development. While grains of brown cultivars showed the presence of phytoene, lycopene, lutein, β-carotene and zeaxanthine at milky stage, the content of each of these metabolites showed marked decrease with progressing grain development. Grains of the brown cultivars accumulated more lutein than β-carotene and zeaxanthine at all the three stages of development. In comparison to white and brown cultivars, grains of purple cultivars accumulated much higher levels of phytoene, lycopene, β-carotene, lutein, and zeaxanthin. The content of each of these carotenoid intermediates showed a progressive increase with grain development till maturity (Table 1, Fig. s1). In contrast to brown cultivars, grains of purple cultivars showed higher level of β-carotene and zeaxanthine than lutein.

**Table 1:** Content of phytoene, lycopene, lutein, β carotene and zeaxanthin (µg gm^−1^ fresh weight) in the whole grain of six different cultivars/accessions representing white, brown and purple accessions/cultivars of rice from North-East India.

Accessions	Pericarp color	Days After Pollination	Phytoene	Lycopene	Lutein	Β-carotene	Zeaxanthine
IC-583122	White	15DAP	nd	nd	nd	nd	nd
		25DAP	nd	nd	nd	nd	nd
		40 DAP	nd	nd	0.06 ± 0.02	nd	nd
IC-558319	White	15DAP	nd	nd	nd	nd	nd
		25DAP	nd	nd	nd	nd	nd
		40 DAP	nd	nd	nd	nd	nd
IC-540274	Brown	15DAP	5.43 ± 0.97	9.32 ± 2.40	14.00 ± 1.20	10.00 ± 1.00	14.00 ± 0.50
		25DAP	4.20 ± 0.12	6.63 ± 2.20	9.44 ± 2.00	5.60 ± 1.40	4.66 ± 0.30
		40 DAP	3.50 ± 0.10	3.4 ± 0.40	6.36 ± 0.80	2.01 ± 1.60	2.10 ± 0.90
IC-558324	Brown	15DAP	2.43 ± 0.60	7.66 ± 0.30	8.00 ± 1.30	7.00 ± 1.40	9.00 ± 2.00
		25DAP	1.26 ± 0.40	4.11 ± 0.40	4.4 ± 2.00	4.60 ± 1.20	7.43 ± 3.20
		40 DAP	0.66 ± 0.20	2.01 ± 0.20	3.10 ± 0.60	0.98 ± 0.30	1.64 ± 0.30
LR 26	Purple	15DAP	10.43 ± 0.06	14.66 ± 0.30	16.00 ± 3.00	29.97 ± 3.00	18.22 ± 2.00
		25DAP	15.43 ± 0.50	16.061 ± 1.00	23.42 ± 4.00	34.12 ± 6.00	20.43 ± 0.43
		40 DAP	18.36 ± 3.00	17.32 ± 4.00	28.89 ± 3.20	49.16 ± 5.00	34.65 ± 4.60
LR 27	Purple	15DAP	10.83 ± 1.00	8.43 ± 0.50	16.43 ± 9.00	10.00 ± 0.80	14.22 ± 0.80
		25DAP	13.43 ± 1.08	9.66 ± 3.20	24.32 ± 4.00	13.42 ± 0.50	15.43 ± 1.00
		40 DAP	14.86 ± 2.20	10.32 ± 2.30	27.16 ± 3.00	28.69 ± 4.00	15.65 ± 2.00

### Whole transcriptome sequencing

After removal of low quality and primer/adapter contaminated reads, we obtained an average of 103,676,662 million high-quality reads from the raw RNAseq data. Mapping of the fragments to msu7 genome with “Tophat2 version 2.0.11” revealed an overall alignment of 83.54% (Table s2). Out of the total number of transcripts identified, 42,427 transcripts were found to be expressed with a sensitivity index of ReadCount >=0. Sequence analysis revealed a total of 31,640 known protein-coding genes apart from lincRNAs, pseudogenes and other types of long RNAs. Transcript type distribution revealed 74.0% sequences as already known transcripts, 22.0% as modified known transcripts and 2.7% as novel intergenic transcripts.

### Transcript distribution at different developmental stages

**Figure 2:**
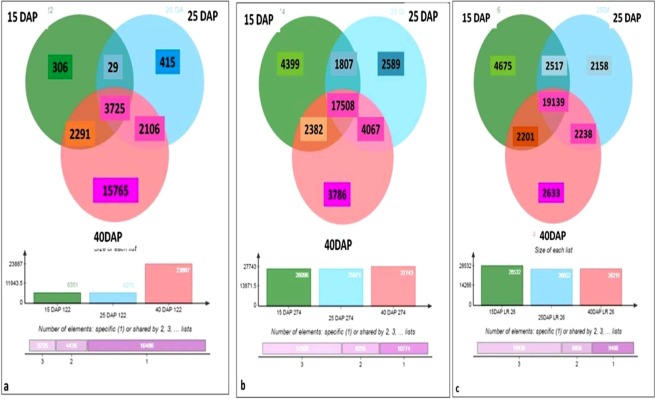
Venn diagram showing the genes expressed in whole grains of white (IC-583122), brown (IC-540274) and purple (LR 26) cultivars of rice from North-East India harvested at milky (15 DAP), doughing (25 DAP) and mature (40 DAP) stages of development. (**a**) genes expressed in IC-583122 at 15 DAP, 25 DAP and 40 DAP. (**b**) genes expressed in IC-540274 at 15 DAP,25 DAP and 40 DAP. (**c**) genes expressed in LR 26 at 15 DAP, 25 DAP and 40 DAP.

Transcript distribution at different stages of grain development in each of the three cultivars of rice is given in Fig. 2. Grains of IC-583122 showed co-expression of 3,725 transcripts at all the three stages of grain development. Transcript distribution analysis revealed 29 co-expressed transcripts at milky and doughing stages. On the other hand, 2,106 transcripts showed co-expression at doughing and mature stages and 2,291 transcripts showed co-expression at milky and mature stages of grain filling. The number of developmental stage-specific genes expressed at milky, doughing and mature stages was 306, 415 and 1,5765, respectively (Fig. 2a). On the other hand, grains of IC-540274 showed co-expression of 17,508 genes at all the three stages of grain development. Data analysis revealed co-expression of 1,807 genes at milky and doughing, 4,067 genes at doughing and mature and 2,382 genes at milky and mature stages of grain development. The number of developmental stage-specific genes expressed at milky, doughing and mature stages was 4,399, 2,599 and 3,786, respectively (Fig. 2b). Transcript distribution analysis revealed co-expression of 19,139 genes at milky, doughing and mature stages in the purple cultivar, LR 26. Whereas 2,517 genes showed co-expression at milky and doughing stages, 2201 genes showed co-expression at doughing and mature stages. 2,238 genes showed co-expression at milky and mature stages in this cultivar. While 4,675 transcripts were specific to milky stage, 2,158 and 2,633 genes were found to be expressed specifically at doughing and mature stages, respectively (Fig. 2c).

Out of the total of 1,12,495 genes designated under different core categories, 55,659 genes were functionally annotated to biological process, 27,490 genes were annotated to cellular processes and 29,346 genes were annotated to molecular functions. 169 genes showed unique ontology.

Within the biological process, the top 5 GOs were cellular process (13.4803%), metabolic process (13.06%), biosynthetic process (8.73%), nucleic acid metabolism (6.76%) and stress responses (6.09%). In the cellular processes category, the top 5 GOs included membrane (14.06%), plastid (12.55%), plasma membrane (11.61%), nucleus (9.92%) and cytosol (8.65%).

The top 5 GOs identified under the category of molecular functions included binding (13.01%), catalytic activity (12.52%), protein binding (12.26%), hydrolase activity (9.83%) and nucleotide binding (8.83%) (Table s3).Mapping of genes to Kyoto Encyclopedia of Genes and Genome database (http://www.genome.ad.jp/kegg/) lead to assignment of 1,180 genes to 121 KEGG pathways. Amongst these, the top 5 pathways with greatest representation of genes included metabolic pathways (18.13%), biosynthesis of secondary metabolites (10%), plant hormone signal transduction (2.9%), carbon metabolism (2.6%) and ribosomes (2.6%) (Table s3). We also carried out GO analysis of novel transcripts and highly expressed genes using the WEGO (Table s4). Gene annotation with Database of Rice Transcription Factors as well as Plant Transcription Factors identified 1,284 TFs belonging to 56 families in the transcriptome. These included NAC (8.48%), ERF (8.41%), bHLH (7.94%), MYB (8.9%), WRKY (6.15%), C2H2 (6.07%) and bZIP (5.37%) families (Table s3).

### Differentially expressed genes

**Figure 3:**
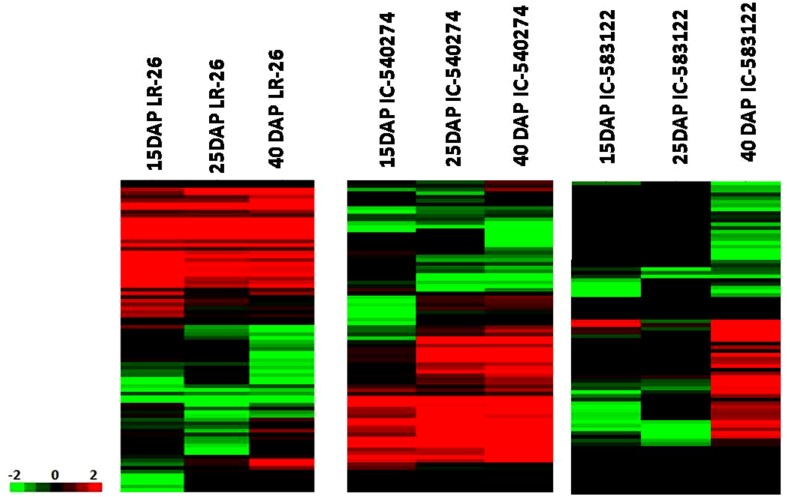
Comparative hierarchical cluster analysis of the differentially expressed genes from whole grains of white (IC-583122), brown (IC-540274) and purple (LR 26) cultivars of rice from North-East India harvested at milky (15 DAP), doughing (25 DAP) and mature (40 DAP) stages of grain filling. Color bars show the corresponding scale for log2 fold change in expression.

**Figure 4:**
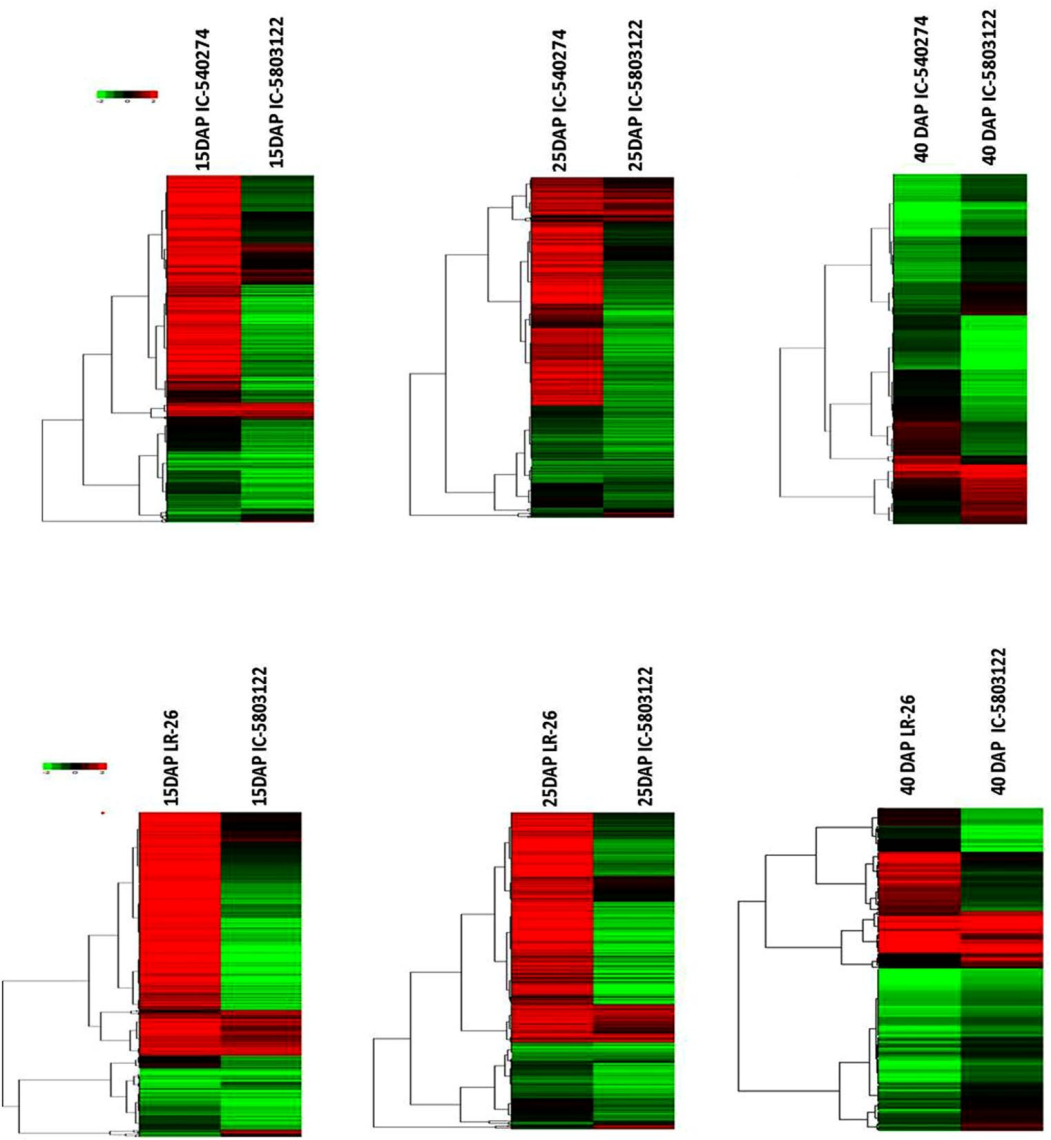
Comparative hierarchical cluster analysis between the white and pigmented cultivars at three developmental stages. (**a**) Pairwise comparison at 15DAP, 25DAP and 40DAP between IC-583122 and LR 26 (**b**) Pairwise comparison at 15DAP, 25DAP and 40DAP between IC-583122 and IC-540274. Color bars show the corresponding scale for log2 fold change in expression.

We used DESeq to estimate differential gene expression as a function of grain development in white (IC-583122), brown (IC-540274) and purple (LR 26) cultivars of rice harvested at milky (15 DAP), doughing (25 DAP) and mature (40 DAP) stages of grain development. Grains of white rice showed 531 DEGs between milky and doughing stages. Out of these 284 genes showed upregulation and 247genes showed down regulation. On the other hand, the number of DEGs between doughing and mature stages was 14,350. Out of these 6,864 genes were upregulated and 7,502 were down regulated. Grains of the brown cultivar showed 2,236 DEGs between milky and doughing stages. Out of these 1,180 genes were upregulated and 1,056 were down regulated. On the other hand, the number of DEGs between doughing and mature stages was 9,427, out of which 4,607 were upregulated and 4,820 were down regulated. The purple cultivar showed differential expression of 2,096 genes between milky and doughing stages of grain development. Out of these, 1,097 genes were upregulated and 999 genes were down regulated. Grains of the purple cultivar showed 8,690 DEGs between doughing and mature stages. Out of these 4,519 genes showed upregulation and 4,171 showed down regulation (Fig. 3). We could observe significant differences in the expression profiles of genes across the cultivars for each developmental stage. While 1,194 transcripts showed differential expression at milky stage between white and purple cultivar, 846 transcripts showed differential expression between the white and brown cultivar for the same stage of development. While the number of differentially expressed transcripts at doughing stage between the white and purple cultivars was 681, the number of differentially expressed transcripts at doughing between white and brown cultivars was 793. The number of differentially expressed transcripts detected at maturity between the white and purple cultivar and between the white and brown cultivar was 147 and 1,437, respectively (Fig. 4).

### Expression profiling of the genes involved in carotenoid biosynthesis

**Figure 5:**
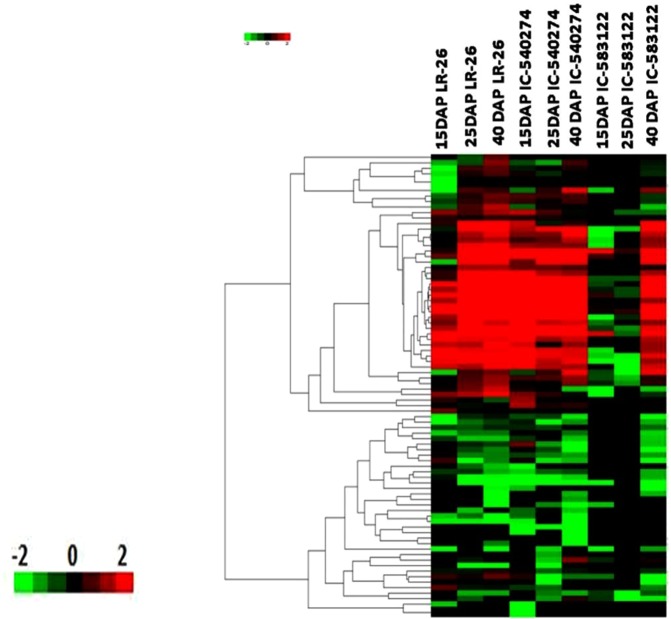
Hierarchical cluster analysis revealing gene expression of carotenoid biosynthetic genes from whole grains of white (IC-583122), brown (IC-540274) and purple (LR 26) cultivars of rice from North -East India harvested at milky (15 DAP), doughing (25 DAP) and mature (40 DAP) stages of grain development. Color bars at the bottom of each of the heat maps show the corresponding scale for log2 fold change in expression. Heat maps were generated for which weighted average linkage method and Pearson correlation distance metric were used.

RNASeq derived data revealed marked differences in the expression profiles of phytoene synthase1 (LOC*_Os*12g43130), phytoene desaturase (LOC*_Os*02g09780), lycopene β-cyclase (LOC*_Os*02g09750) and lycopene ε-cyclase (LOC*_Os*01g39960) between the white, brown and purple cultivars at each stage of grain development (Table s5). In comparison to the white and brown cultivars, grains of the purple cultivar (LR 26) showed higher expression of PSY1, PDS and β-LCY at all the stages of development. Grains of white cultivar (IC-583122) showed no significant changes in the expression of PSY1 and PDS during transition from milky to doughing stage, however, both the genes showed a nearly 2 fold increase in expression between doughing and mature stages (Table s5). While the grains of the white cultivar did not show any expression of β-LCY at any stage of development, expression of ε-LCY was detected only at maturity in this accession. The expression of ε-LCY showed a near 2.5 fold increase during the transition from doughing to mature stage of grain development. The grains of brown cultivar (IC-540274) showed a progressive decrease in expression of PSY1 and PDS during transition from milky stage to maturity. On the other hand, grains of the purple cultivar (LR 26) showed a significant increase in expression of both these genes with progressing grain development. While the grains of brown cultivar showed higher expression of ε-LCY than β-LCY at all the stages of development, those of purple cultivar showed higher expression of β-LCY than ε-LCY at all stages of development (Table s5, Fig. 5).

### Co-expression networking amongst pathways/processes

**Figure 6:**
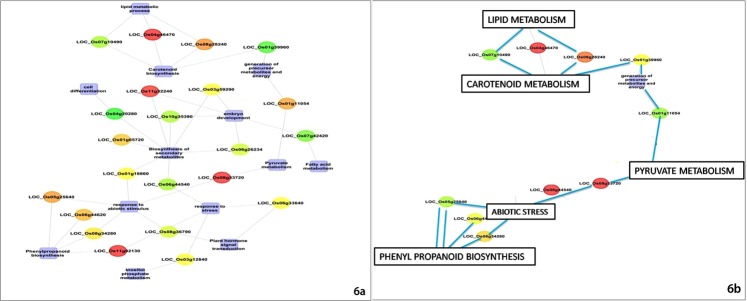
Functional gene network developed from the transcriptome data of grains studied in present investigation. (**a**) Pathways co-expressing with the carotenoid biosynthetic pathway: pyruvate metabolism, lipid metabolism, secondary metabolism, cell differentiation, inositol phosphate metabolism, embryo development, phenyl propanoidpathway, MEP pathway and abiotic stress. (**b**) Pathways/processes directly linked with the carotenoid metabolic pathway: pyruvate metabolism, lipid metabolism, phenyl propanoid pathway, MEP pathway and abiotic stress.

We developed a nested network between the differentially expressed genes involved in phenyl propanoid biosynthesis, pyruvate metabolism, fatty acid metabolism, carotenoid biosynthesis and plant hormone signal transduction using “CytoScape tool”. The network revealed a high degree of interaction between pyruvate metabolism, lipid metabolism, secondary metabolism, cell differentiation, inositol phosphate metabolism, embryo development, phenyl propanoid biosynthesis, isoprenoid biosynthesis, abiotic stress and carotenoid biosynthesis (Fig. 6a). Coexpression networking revealed a strong interaction between pyruvate metabolism, lipid metabolism, phenyl propanoid biosynthesis, isoprenoid biosynthesis, abiotic stress and carotenoid biosynthesis (Fig. 6b). While the genes coding for enzymes involved in pyruvate biosynthesis such as hexokinase (LOC*_Os*05g45590.1), fructose1,6 –bisphosphatase aldolase (LOC*_Os*01g67860.1), 6-phosphofructokinase2 (LOC*_Os*04g39420.1), glyceraldehyde-3-phosphate dehydrogenase (LOC*_Os*06g45590.1) and pyruvate kinase (LOC*_Os*03g46910.1) did not show any significant expression in the grains of white cultivar at the milky and doughing stages, there was an increase in the expression of these genes in grains of this cultivar during transition from doughing stage to maturity (Table S6a). On the other hand grains of the brown cultivar showed a relatively higher expression of these genes at milky stage of development. The expression of each of these gene, however, showed a progressive decrease during transition of the grains from milky stage to maturity (Table s6a). Compared to the white and brown cultivars, grains of purple cultivar (LR 26) showed marked increases in the expression of hexokinase (LOC*_Os*05g45590.1), fructose1,6 –bisphosphatase aldolase (LOC*_Os*01g67860.1), 6-phosphofructokinase2 (LOC*_Os*04g39420.1), glyceraldehyde-3-phosphate dehydrogenase (LOC*_Os*06g45590.1) and pyruvate kinase (LOC*_Os*03g46910.1) with progressing grain development from milky stage to maturity (Table s6a). Grains of the purple cultivar also showed higher expression of 9-cis-epoxycarotenoid dioxygenase (LOC *_Os*02g47510, LOC*_Os*12g42280) (Table s6a). Co-expression networking also revealed a strong positive relationship between the expression profiles of genes involved in carotenoid biosynthesis and genes coding for geranylgeranyl transferase type II, glutathione S-transferase, DnaJ and SET domain containing proteins (Table s6b). Compared to the brown and white cultivars, grains of the purple cultivar showed significantly higher expression of genes coding for geranylgeranyl transferase type II (LOC*_Os*11g32090), glutathione S-transferase (LOC*_Os*10g38489), chaperone DnaJ10 (LOC*_Os*08g41110), DnaJ domain-containing protein (LOC*_Os*05g48810) and SET domain-containing protein (LOC*_Os*08g14660). The fold change in expression of these genes correlated strongly with changes in the level of carotenoid intermediates, especially during grain maturation (Table s6b).

**Figure 7:**
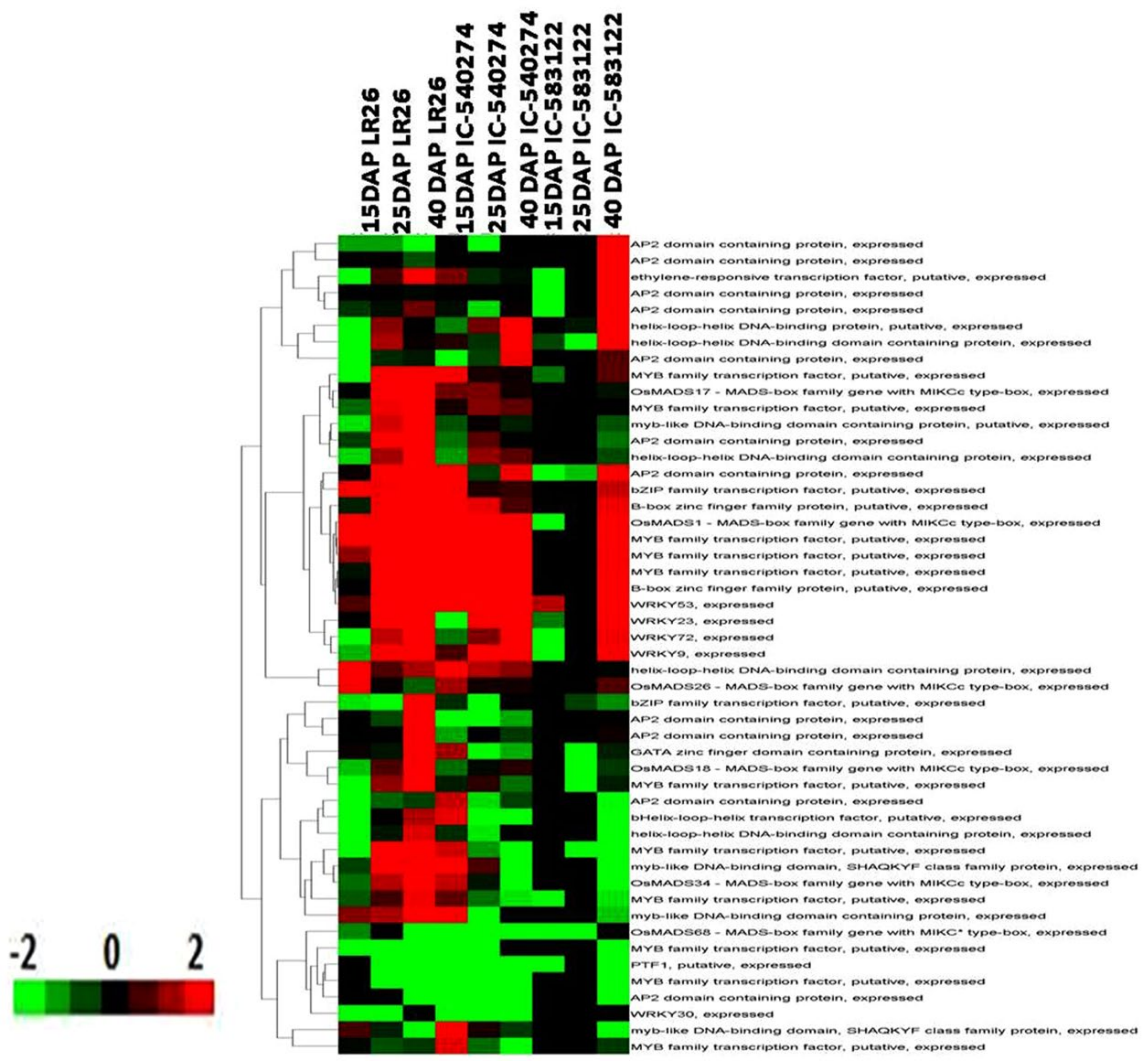
Heat map of the correlation analysis of differentially expressed transcription factors from whole grains of IC-583122, IC- 540274 and LR 26 with the carotenoid biosynthetic genes. The rice grains were harvested from North-East India at 15 DAP, 25 DAP and 40 DAP. Color bars at the bottom of each of the heat maps show the corresponding scale for log2 fold change in expression.

Transcript abundance covariance, carried out between selected genes of the carotenoid biosynthesis pathway and transcription factors revealed the involvement of nineteen families of transcription factors with genes involved in carotenoid biosynthetic pathway (Table s7). Whereas light specific TFs belonging to WRKY, MYB, bHLH and zinc finger families showed higher expression at all stages of grain development in the purple and brown cultivars, their expression was registered only at maturity in the grains of white cultivar (Fig. 7). On the other hand, AP-2 and AP2/ERF, which are negative regulators of carotenoid metabolism, showed either no expression or down regulation of expression in grains of purple and brown cultivar at all stages of development. The expression of these TFs however, showed a nearly 3 fold increase during grain maturation in white rice (Fig. 7). Data analysis also revealed a strong correlation between MADS26 (LOC*_Os*08g02070), and R2R3MYB (LOC_Os06g10350) transcription factors with expression of carotenoid biosynthetic genes (Table s6c). Whereas *Os*MADS26 and R2R3MYB showed no expression at milky and doughing stages in grains of white cultivar, the purple cultivar showed significantly high expression of both these genes at all stages of grain development; the fold changes in their expression correlated strongly with the expression profiles of genes involved in carotenoid biosynthesis (Table s6c).

### Validation of differential gene expression

qRT-PCR of selected genes listed in Table S1 was performed to validate RNASeq based transcriptome profile. A comparative analysis of the selected genes revealed a high degree of consistency between transcript abundance determined by qRT-PCR and RNAseq (Table S8).

## Discussion

Secondary metabolites, such as anthocyanins, flavonols, proanthocyanidins and carotenoids, play important roles in plant development. Even though the basic biosynthetic pathways leading to synthesis of the core secondary metabolites have been well-characterized, the mechanisms underlying various regulatory controls in carotenoid biosynthesis continue to be a matter of debate. Except for a small amount of lutein in the pericarp of mature grains of white rice, we could not detect any other carotenoid intermediate in the grains of white cultivars at any stage of development. On the other hand, mature grains of purple cultivars accumulated significant levels of phytoene, lycopene, β-carotene, lutein, and zeaxanthin with LR 26 accumulating upto 49.16 ± 5 µg of β -carotene, 28.89 ± 3.2 µg lutein and 34.65 ± 0.6 µg of zeaxanthin gm^−1^ grain fresh weight. Similar reports on high level of carotenoid intermediates in the grains of black rice has been reported by Frei and Becker^13^ and Kim *et al*.^14^. Schaub *et al*.^21^ have attributed the absence of carotenoids in white rice to the absence of PSY1 transcripts and limited availability of precursor substrates necessary for PDS/ZDS activity in the endosperm. While the grains of white cultivar did not show any expression of PSY1 at milky and doughing stages, expression of this gene was recorded in mature grains of this cultivar. Accumulation of marginal levels of lutein in mature grains of white cultivar can be ascribed to expression of PSY1 gene in grains of this cultivar at maturity.

Irrespective of the cultivar, the number of DEGs recorded in grains at doughing stage and maturity was much higher than that recorded between milky and doughing stages. Enrichment analysis at FDR < 0.05 revealed higher expression of genes involved in to signal transduction, amino acid, lipid and energy metabolism, nucleic acid replication/processing and transcriptional regulation at milky stage. On the other hand, genes involved in protein biosynthesis showed higher expression during doughing stage and those involved in storage protein accumulation, starch metabolism and secondary metabolism showed higher expression during grain maturation. These results clearly indicate differential patterning of metabolic pathways during transition of grains from milky stage to maturity. Similar expression kinetics of DEGs has been reported in other cultivars of rice^22^ and *Arabidopsis*^23^. Interestingly, our results revealed a concomitant upregulation of the genes involved in ABA [NCEDs (LOC*_Os*12g42280, \LOC*_Os*02g47510), aldehyde oxidase2 (LOC*_Os*03g57720)] and GA [ent-kaurene synthase A (LOC*_Os*04g36760), KA01 (LOC*_Os*10g23160), *Os*GA20ox1 (LOC*_Os*03g42130.1), GA2OX7 (LOC*_Os*04g33360, LOC*_Os*04g44150)] biosynthesis in the grains during their development. Such a simultaneous upregulation of genes involved in ABA and GA_3_ biosynthetic pathways during grain maturation has also been reported in seeds of *Hordeum vulgare*^24^. Sreenivasulu *et al*.^24^ suggested that GA not only played an important role in triggering major proteinases during early seed germination but also acted in mobilization of distinct reserve polysaccharides during grain maturation. GA_3_ is known to cause endosperm tissue weakening at the micropylar end by modulating expression of genes encoding cell wall remodeling enzymes^25^. In addition, the scutellum of the embryo secretes GA to the aleurone layer of rice grains which is devoid of starch molecules. GA induces the secretion of amylases and proteases that break down the stored starch and proteins in the starchy endosperm, making free sugars and amino acids available for other metabolic processes^25^. In barley, differentiation of nucellar projection, which forms a link to filial organs was shown to be modulated by a shift from lower to higher GA/ABA ratios during grain maturation^26^. It was suggested that the change in GA/ABA ratios was a prerequisite for the formation of a differentiation gradient which is essential for ordered assimilate transfer through the nucellar projection.

Our results showed a positive correlation in expression profile of PSY1 with differences in the level of phytoene and other carotenoid intermediates between the white, brown and purple cultivar, thereby indicating the significance of PSY1 in conditioning carotenoid content and composition in different cultivars of rice. Miller *et al*.^27^ have made similar observations on the correlation between PSY1 expression and total carotenoid content in other cultivars of rice. Whereas the ratio of lutein to β-carotene in milky and mature grains of brown cultivar was 1.4 and 3.16, respectively, it was 0.53 and 0.58 at corresponding stages of in the grains of purple cultivar. The difference in the ratio of lutein to β-carotene in grains of brown and purple cultivars is most likely due to the differences in the expression of β-LCY and ε-LCY in these cultivars. Differences in the expression of β-LCY and ε-LCY in grains of brown and purple cultivars determines a preferential flux of lycopene towards the synthesis of either the β-branch carotenoids or ε-branch carotenes. The higher expression of β-LCY in grains of purple cultivar would determine the flux of lycopene towards β-branch carotenoids in this cultivar. Such a flux of lycopene towards synthesis of β,ε-carotenoids or β,β-carotenoids by activities of β-LCY and ε-LCY has been well illustrated in crops belonging to Poaceace by Bai *et al*.^28^ and Kandianis *et al*.^29^. Bai *et al*.^28^ has ascribed the differential pattern of carotenoid expression in maize embryo and endosperm to differential expression of genes encoding lycopene β-cyclase and lycopene ε-cyclase in these two tissues.

Coexpression networking clearly indicated a strong relationship between pyurvate metabolism and carotenoid biosynthesis in the grains. Cultivars with higher carotenoid levels in their grains also showed higher expression of genes involved in pyruvate metabolism. Such relationships between enahanced pyruvate flux and carotenoid accumulation in the grains have also been reported in rice^3^, maize^30^ and *Arabidopsis*^31^. Additionally, quantitative proteome analysis carried by Chen *et al*.^32^ in grains of black rice (*O*. *sativa* L. *indica* var. SSP) also revealed high expression of enzymes belonging to carbohydrate metabolism as well as high sugar levels in the grains of black rice. It is thus possible that higher expression of genes involved in pathways lying upstream of carotenoid metabolism in the purple cultivar (LR 26) makes enhanced pool of metabolites available to the isoprenoid biosynthesis pathway for synthesis of carotenoids. Grains of purple cultivar also had higher expression of 9-cis-epoxycarotenoid dioxygenases that provide the precursors for ABA biosynthesis. This correlates with the observations of Meier *et al*.^31^, Lindgren *et al*.^33^ and Barickman *et al*.^34^ who have argued for a strong relationship between ABA biosynthesis and carotenoid levels in plants. We opine that both upstream pathways supplying carotenoid precursors as well as downstream pathways that metabolize or sequester carotenoids to deplete the carotenoid pool have an important bearing on the rate of carotenoid biosynthesis in rice. Purple rice also showed a consistently higher expression of transcripts coding for DnaJ10 and DnaJ domain-containing proteins. OR encodes a Cys-rich DnaJ-like zinc finger domain-containing protein^35^. Li *et al*.^35^ and Lu *et al*.^36^ have observed that DnaJ-like zinc finger domain-containing protein induced chromoplast differentiation thereby enhancing the storage sink for carotenoids. Overexpression of Or gene from cauliflower has been reported to enhance accumulation of carotenoids in potato tubers by inducing differentiation of chromoplasts with enhanced sink strength^37^. The high expression of transcripts for DnaJ10 and DnaJ domain-containing proteins in grains of purple rice could be inducing enhanced sink strength for carotenoid sequestration in this cultivar. Our results also showed higher expression of transcripts coding for SET domain-containing protein in grains of purple cultivar. The fold change in expression of SET domain-containing protein correlated with changes in the level of carotenoid intermediates, especially so during grain filling, thereby indicating a role for these proteins in regulating carotenogenesis in plants. Cazzonelli *et al*.^38^ had earlier demonstrated the essential requirement of histone methyltransferase (SET DOMAIN GROUP 8, *SDG8*) that methylates histone H3 on Lys 4 and/or 36 (H3K4 and H3K36) for maintenance of permissive expression of carotenoid isomerase (CRTISO) in *A*. *thaliana*. CRITSO is required for converting *cis* form of lycopene to *trans* form which is essential for cyclization to beta-carotene. Mutations in SDG8 (*ccr1*) were shown to result in a significant reduction in transcript levels of CRTISO, leading to the accumulation of cis –isomers of carotenoid intermediates^38^.

Correlation analysis between the genes involved in carotenoid biosynthesis and various families of transcription factors revealed higher expression of light specific transcription factors in the grains of purple and brown cultivar than that in white rice. Similar observations on the involvement of WRKY, MYBs, bZIPs and bHLHs (PIFs) in transcriptional regulation of genes involved in carotenoid biosynthesis have been made by Mohanty *et al*.^39^ and Liang and Jiang^40^. Whereas information on specific transcription factors controlling the expression of carotenoid biosynthetic genes is available for horticultural crops, information on such proteins in controlling carotenogenesis in cereals is sketchy. Our results show a strong correlation between the carotenoid content in the different rice cultivars and the expression of *Os*MADS26 and R2R3MYB genes. Although *Os*MADS26 has been reported to manifest phenotypes related to pigment accumulation in rice^41^ and R2R3MYB has been regarded as candidate gene for hull pigmentation in rice^42^, evidences of involvement of MADS and R2R3 MYB have reported in controlling carotenoid accumulation in tomato^43^ and *Mimulus lewisii*^44^ flowers respectively.

**Figure 8:**
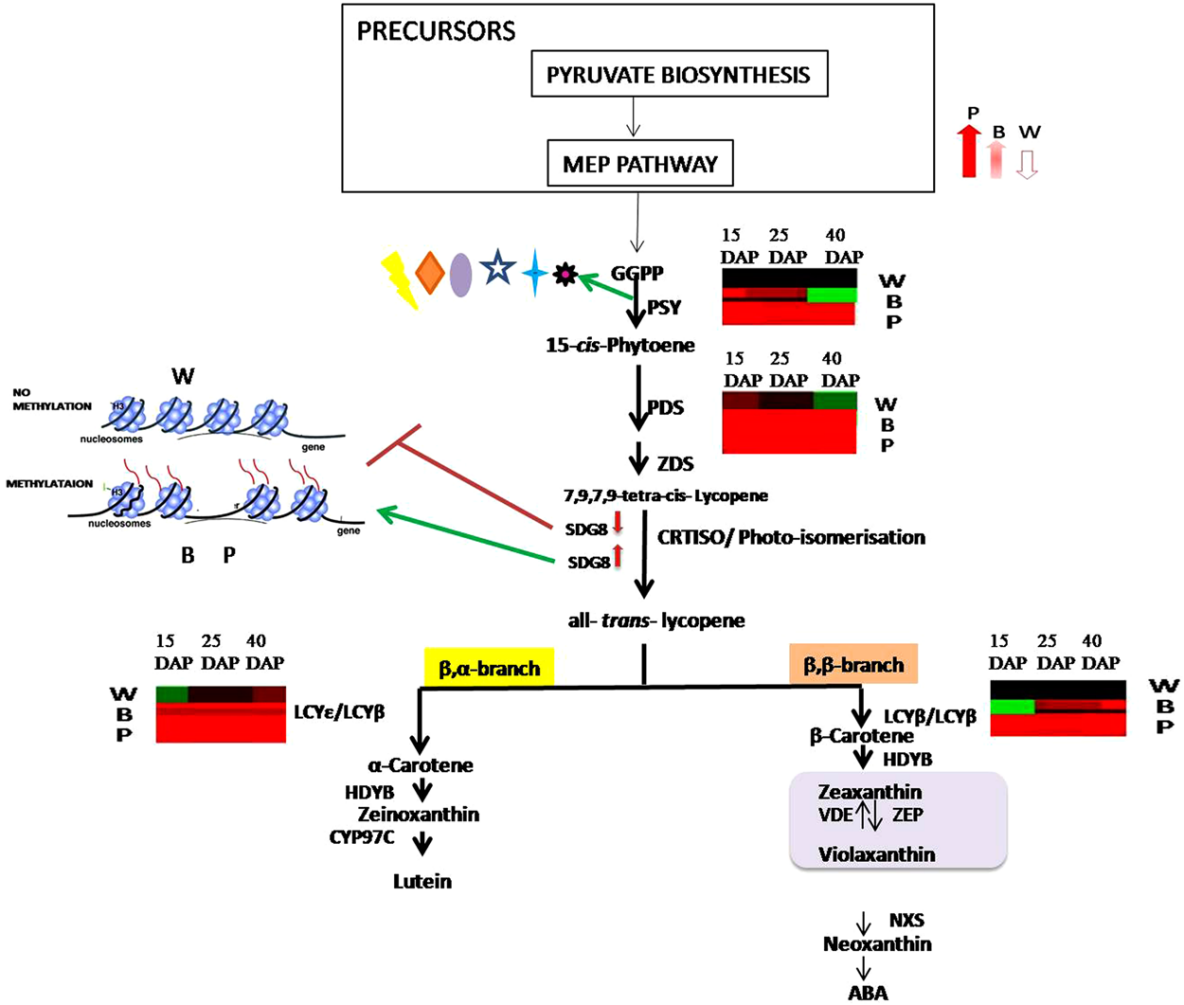
Pictorial representation of the carotenogenic regulators modulating in different varieties of rice. Red upward pointing arrows indicates gene expression positively correlated with carotenoid biosynthesis; Red downward pointing arrows indicate gene expression negatively correlated with carotenoid biosynthesis. Green arrows indicates factors that postively regulates gene expression; Red arrow with block, indicates factors that negatively regulate gene expression. Transcription factors and light are represented in different symbols. PSY: phytoene synthase, PDS: phytoene desaturase, βlcy: β-lycopene cyclase, ε-LCY: ε lycopene cyclase, BCH: βcarotene hydroxylases. W: White, B: brown, P: Purple (Expression of genes has been represented in form of heat map (red-upregulation, green –downregulation and black-no regulation).

Our investigation points towards a multifaceted regulation of carotenoid biosynthesis in rice. Besides the transcriptional regulation of genes such as PSY1,PDS, β-LCY and ε-LCY, additional regulatory controls leading to higher carotenoid content in purple rice include pathway crosstalk between the upstream channels (pyruvate metabolism, MEP pathway), as well as the downstream pathways including carotenoid sequestration and ABA biosynthesis. Co-expression analysis indicates a positive relationship between expression of genes involved in carotenoid biosynthesis and light specific transcription factors such as WRKY, MYBs, bZIPs and bHLHs, glutathione S-transferase, Or protein and SET DOMAIN containing protein genes, thereby suggesting a role of these factors in regulating carotenogenesis in rice (Fig. 8). High expression of Or protein encoding gene and SET DOMAIN in the purple cultivar indicates a role of sequesteration site and epigenetic factors such as SDG8-mediated histone methylation of regulatory domain of CRTISO in regulating rice carotenogenesis.

## Supplementary information

Figs1, Table s1, Table s2

Table s3

table s4

Table s5

Table s6

Table s7

Table s8
