# Supplementary material for: Comparative transcriptomics approach in elucidation of carotenoid biosynthesis regulation in grains of rice (Oryza sativa L.)
Source: Sci Rep. 2019 Feb 7;9:1631. doi: 10.1038/s41598-018-38233-8 (PMC6367445; doi:10.1038/s41598-018-38233-8)
Supplement: Supplementary file 1 — Figs1, Table s1, Table s2 [file 41598_2018_38233_MOESM1_ESM.pdf]

Comparative transcriptomics approach in elucidation of carotenoid biosynthesis regulation in grains of rice (*Oryza sativa* L.)

Upasna Chettry1, Nikhil K. Chrungoo1* and Kirti Kulkarni2
Department of Botany, North Eastern Hill University, Shillong-793022 India

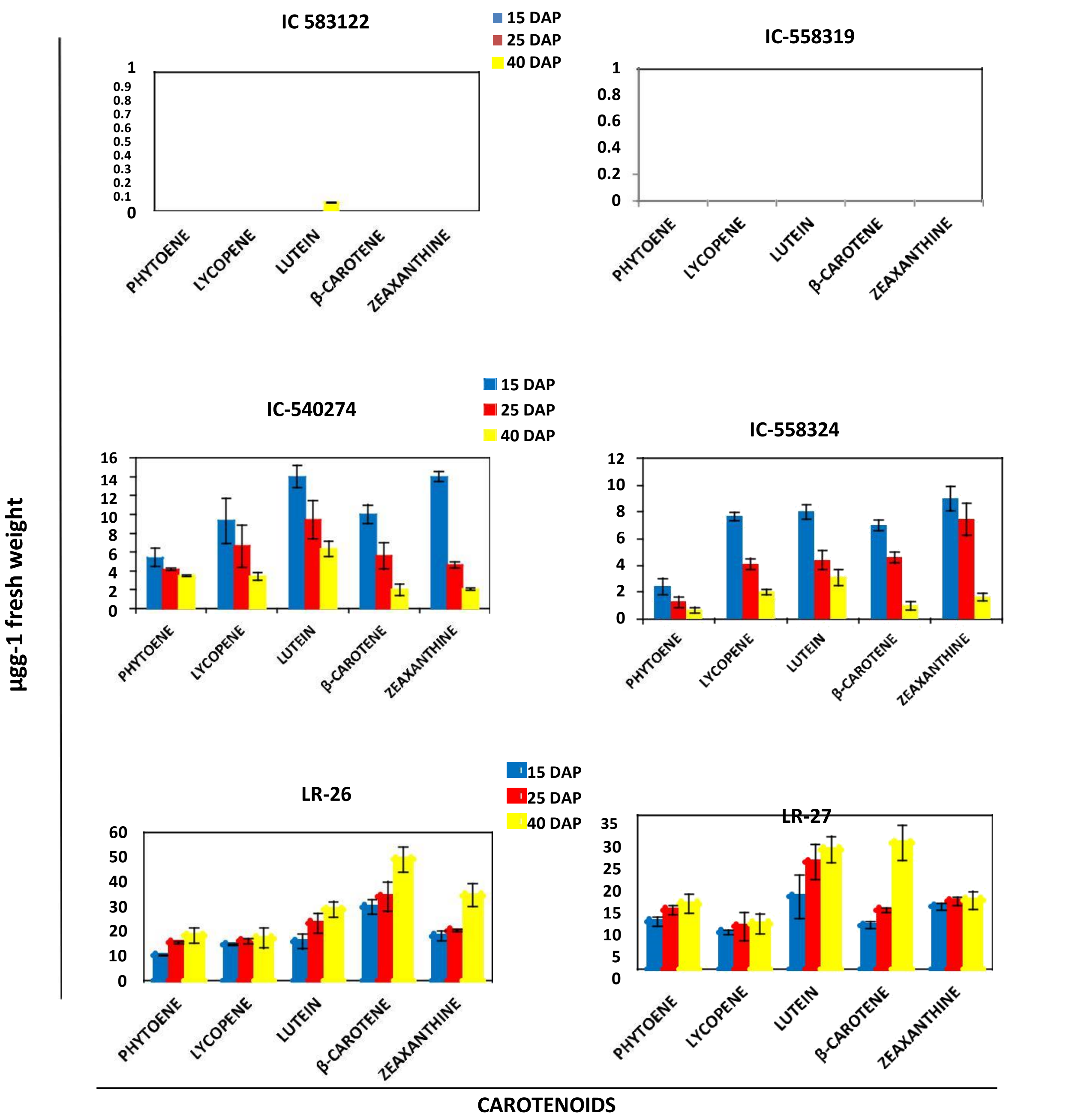

Fig. s1:Carotenoid profile of whole grains of six different cultivars/accessions representing white (IC-583122, IC-558319), brown (IC-540274, IC-558324) and purple (LR 26, LR 27) cultivars of rice from North-East at milky (15 DAP), doughing (25 DAP) and mature (40 DAP) stages of development. Vertical lines in each bar represent ± SE at P0.05.

Table s1: Name, nucleotide sequence, G+C content (%) and T_m of oligonucleotide primers used in the present study for amplification of Phytoene desaturase, Phytoene synthase, β-carotene hydroxylase, β-lycopene cyclase, ε-lycopene cyclase, Hexokinase, Pyruvate kinase, Fructose-1,6 bisphosphatase, Geranylgeranyl transferase, 9-cis-epoxycarotenoid dioxygenase 1, MADS26, and MYB family transcription factor from cDNA synthesized from mRNA isolated from grains of white (IC-583122), brown (IC-540274) and purple (LR 26) cultivars of rice.

**Target gene**	**Primer name**	**Nucleotide sequence**	**G+C content (%)**	**T_m ^oC**	**Reference**
Phytoene desaturase	PDS F	5' ATGATCCAAACCGTTCAATGCT3'	41.0	57.6	Present study
	PDS R	5' CTCCGTCCAACCCATTCCT 3'	43.0	57.7	
Phytoene synthase	PSYF	5' GACGAATATTCTCAGAGACG3'	42.4	59.6	Present study
	PSYR	5' ACTTTCCCTCTGAATATGTC3'	37.0	59.3	
β-carotene hydroxylase	BCHF	5'CCCTACTTCCGCCGAGTTG3'	45.1	55.3	Present study
	BCHR	5'CCTTCGAACTTGTCCGTGTGA3'	46.9	56.5	
β-lycopene cyclase	β LCYF	5'GGTGCCTCGTCCAGTACGA3'	43.6	55.4	Present study
	β LCYR	5'ATGAACAGCATCTTGTCGATGT3'	50.9	54.7	
ε-lycopene cyclase	εLCY F	5'GCAACTCCTCGTGCAGCAA3'	55.7	55.5	Present study
	εLCY R	5' TGATTGGCCGTGCGTATG3'	46.3	56.0	
Hexokinase	HKF	5' TTATACTGGGAACAGGTACTA3'	43.4	50.0	Present study
	HKR	5'AAGCTTCTTCAGCCATCTTTAATA3'	50.8	53.0	
Pyruvate Kinase	PKF	5' TGGAGTTATGGTAGCACGTG3'	50.1	52.2	Present study
	PKR	5' ATGTTGTGGAAGAAGTTTCT3'	40.8	50.3	
Fructose-1,6bisphosphatase	FBF	5' AAAGGTTGCAGTGATGGCTT3'	45.2	50.0	Present study
	FBR	5'TCAGCAACCAGCGAGCACAC3'	53.7	53.1	
Geranylgeranyl transferase	GTF	5' CGGATGCGAGTTGCTCGATGCA3'	59.2	58.2	Present study
	GTR	5' TTCTCGCGGATCCGGAAT3'	56.1	53.6	
9-cis epoxycarotenoid dioxygenase 1	NCEDF	5' TTCTCGCGGATCCGGAAT3'	56.0	50.3	Present study
	NCEDR	5' TCCCACGCGTTCACCGAGTGC3'	58.3	56.9	
MADS26	MADF	5'GGTACAAGAGTGCTAGTGGCGAAC3'	54.3	59.1	Present study
	MADR	5' CATCCATATCTCTAAGTAC3'	44.9	50.3	
MYB family transcription factor	MYB TF	5' AAGCACGGCGGCTACACCGAAGC3'	58.0	62.4	Present study
	MYB TR	5'CAAGCTCAAGAAGAAAGCC3'	47.9	50.9	

Table s2: Number of raw reads, high quality reads (HQ), low quality reads (LQ) and reads mapped to rice reference sequence (msu7) generated from whole transcriptome sequencing of grains of IC-583122, IC-540274 and LR 26 cultivars of rice from North-East India harvested at 15 DAP, 25 DAP and 40 DAP.

ACCESSION	DAY OF HARVEST	RAW READS	HQ READS	LQ READS	ALIGNED
IC-583122	15 DAP	5,10 Million	4,920 Million (96.54%)	0.1	4876783
IC-583122	25 DAP	1,94 Million	1,789 Million (92.09%)	0.7	1714632
IC-583122	40 DAP	11.71 Million	10.98 Million (93.80%)	0.73	9282051
IC-540274	15 DAP	12.21 Million	11.48 Million (94.01%)	0.73	9256195
IC-540274	25 DAP	14.47 Million	13.68 Million (94.60%)	0.78	11501224
IC-540274	40 DAP	20.85 Million	19.69 Million (94.65%)	1.16	16115375
LR-26	15 DAP	21.36 Million	20.22 Million (94.65%)	1.14	17017906
LR-26	25 DAP	13.71 Million	12.92 Million (94.27%)	0.79	10937834
LR-26	40 DAP	15.56 Million	14.70 Million (94.47%)	0.86	12490526
